# Systematic Review of Sensorineural Hearing Loss Associated With COVID-19 Infection

**DOI:** 10.7759/cureus.19757

**Published:** 2021-11-19

**Authors:** Kelcy M McIntyre, Nicole M Favre, Cathleen C Kuo, Michele M Carr

**Affiliations:** 1 Otolaryngology, University at Buffalo Jacobs School of Medicine and Biomedical Sciences, Buffalo, USA

**Keywords:** sars-cov-2, sudden sensorineural hearing loss, sar- cov-2, sensorineural hearing loss, coronavirus, covid-19

## Abstract

Our objective is to identify novel coronavirus disease 2019 (COVID-19) patients with a diagnosis of sudden sensorineural hearing loss (SSNHL) with an aim to describe possible mechanisms. A systematic review was conducted using PubMed and Google Scholar. Preferred Reporting Items for Systematic Reviews and Meta-Analyses (PRISMA) guidelines were followed^. ^Our search terms included: "Sensorineural Hearing Loss" + "COVID-19" or "Sensorineural Hearing Loss" + "SARS-CoV-2" or "Sensorineural Hearing Loss" + "Coronavirus". Studies that adhered to the inclusion and exclusion criteria were included in the review. Of the 20 articles identified in the initial search, five met the inclusion criteria. The included articles consisted of four case studies and one letter to the editor, with seven total patients analyzed. All patients were COVID-19 positive and exhibited SSNHL, either unilateral or bilateral. Four patients reported tinnitus and two patients experienced vertigo. One patient was treated with hydroxychloroquine and one patient was treated with a variety of medications. Four patients were treated with intravenous and/or oral steroids intended to treat the SSNHL. The current literature describing SSNHL in COVID-19 patients is insufficient to characterize the pattern of hearing loss or advise about the treatment or outcomes. Future studies require a larger database or population study.

## Introduction and background

In December 2019, there was a report of a series of patients experiencing pneumonia thought to be from the zoonotic transmission of a novel virus related to a large seafood market in China [1]. Following similar reports, the World Health Organization declared the novel coronavirus 2019 (COVID-19) a global pandemic on March 11, 2020 [2]. Common clinical symptoms of COVID-19 include fever, cough, and fatigue, but many patients remained asymptomatic or experienced atypical symptoms, some of which are detailed in a paper by Stawicki et al. [3]. Viral infections have been implicated in the past as etiologic agents for some of these atypical symptoms such as neurological symptoms including hearing loss, anosmia, and facial paralysis [4]. Based on multiple cross-sectional studies, the incidence of olfactory dysfunction in COVID-19 patients varies from 33.9% to 68% [4]. In a study by Lechien et al., 12% of patients presented with anosmia as the first symptom [5]. Although facial nerve palsy has rarely been associated with COVID-19, some cases have been identified along with occasional involvement of other cranial nerves [6]. Recently, a small number of patients with COVID-19 and symptoms of hearing loss, vertigo, and tinnitus have been described [7-9]. Therefore, it is imperative that providers be mindful of these rare symptoms to prevent a delay in COVID-19 diagnosis.

Sensorineural hearing loss (SSNHL) is a permanent form of hearing loss resulting from damage to the inner ear or the auditory nerve [10]. SSNHL is defined as a loss of 30 dB or more across at least three contiguous frequencies occurring within 72 hours [11]. Mechanisms that have been proposed to explain how viral infection could lead to SSNHL include viral invasion of the cochlear nerve or fluid spaces, reactivation of latent virus within tissues of the inner ear, or indirect antibody triggering by the virus [12]. COVID-19 has not been fully assessed for its ability to invade the auditory pathways, but previous studies describing virus-associated SSNHL suggest the possibility [13]. 

 In addition to a viral etiology of SSNHL in COVID-19, pharmacologic ototoxicity related to COVID-19 treatment regimens may also be implicated. Specifically, this includes chloroquine and hydroxychloroquine, which are known to be ototoxic [14]. These two drugs have long been used in the treatment of malaria and chronic inflammatory diseases [13]. Studies have reported possible clinical implications for COVID-19 patients treated with hydroxychloroquine, including SSNHL, tinnitus, abnormal gait, and vertigo [15]. The recommended dosage of chloroquine and hydroxychloroquine for patients with COVID-19 is substantially higher than the dosage used for malaria and chronic inflammatory diseases [16]. 

 In this systematic review, we identified COVID-19 positive patients with a diagnosis of SSNHL with an aim to describe possible mechanisms.

## Review

Methods

**Figure 1:**
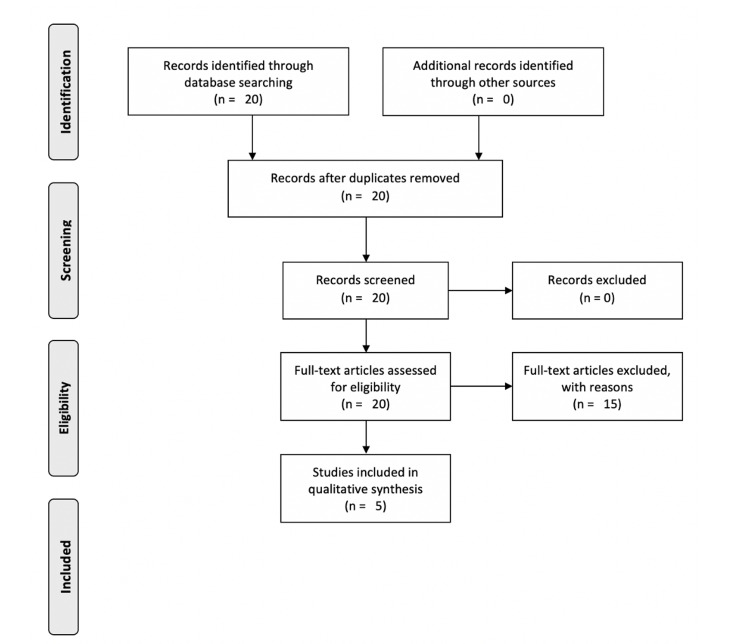
PRISMA Flow Diagram Adapted from: Selçuk, 2019 [17] PRISMA: Preferred Reporting Items for Systematic Reviews and Meta-Analyses

We conducted a systematic review by searching PubMed and Google Scholar without date, geographic location of study, or language restrictions (performed January 2021). Preferred Reporting Items for Systematic Reviews and Meta-Analyses (PRISMA) guidelines were followed (Figure 1) [17]. Our search terms included: "Sensorineural Hearing Loss" + "COVID-19" or "Sensorineural Hearing Loss" + "SARS-CoV-2" or "Sensorineural Hearing Loss" + "Coronavirus". 

To be eligible for inclusion in the analysis the following criteria were required: patients must have a diagnosis of COVID-19 confirmed by polymerase chain reaction (PCR) or must have a diagnosis of SSNHL and the articles were peer-reviewed.

Studies were excluded if: patients did not have a diagnosis of COVID-19 confirmed by PCR or patient did not have a specific diagnosis of SSNHL (including bilateral or unilateral)

Twenty novel articles were discovered by screening titles and abstracts of papers. For articles that appeared to meet the inclusion criteria, the full-text article was reviewed. Two reviewers independently screened the titles and abstracts of articles. Three reviewers reviewed and compiled the data from each article. Any disagreements were discussed among the three reviewers and agreed upon prior to final inclusion. We included the year of publication, country of publication, type of study, patient age, gender, symptoms, COVID-19 treatment, and SSNHL treatment.

Results

Of the 20 articles reviewed in their entirety, five articles met the inclusion criteria. Of those, seven cases were included in the study.

Of the seven cases, four (57%) patients were female and three (43%) patients were male. All of the patients were between the ages of 18 and 45 years. All patients exhibited SSNHL, either unilateral or bilateral. Four (57%) patients reported tinnitus. Two (29%) patients experienced vertigo. One (14%) patient also exhibited the additional findings of aural fullness and MRI findings consistent with intralabyrinthine hemorrhage (Table 1). 

**Table 1:** Demographics and Clinical Manifestations of Patients With SSNHL and Confirmed COVID-19 Diagnosis N: Number of patients; F: Female; M=Male; NR: Not reported; SSNHL: Sudden sensorineural hearing loss; L: Left; R: Right

Author, Year	Country	Type of Study	N	Age (years)	Sex	Otologic, Vestibular or Aural Symptoms	SSNHL Description	COVID Symptoms
Chern et al. 2020 [7]	United States	Case report	1	18	F	Bilateral SSNHL, intermittent bilateral aural fullness, vertigo	R: Pure-tone average of 60 dB, 88% word recognition score L: Pure-tone average of 63 dB, 80% word recognition score	Nausea, vomiting, loss of taste, and olfaction
Kilic et al. 2020 [18]	Turkey	Case series	1	29	M	Right-sided SSNHL	R: Profound high-frequency SSNHL L: No audiogram reported	Fever, cough, headache, myalgia
Lang et al. 2020 [9]	Ireland	Case report	1	30	F	Right-sided SSNHL, tinnitus	R: Profound high-frequency SSNHL L: No audiogram reported	Fever, cough, headache, myalgia
Koumpa et al. 2020 [19]	United Kingdom	Case report	1	45	M	Left-sided SSNHL, tinnitus	Weber test lateralized to the right, negative Rinne test R: No audiogram reported L: Pure tone audiogram - 2, 3, 4, and 6 kHz with hearing thresholds of 65, 75, 75, and 85 dB respectively	Asymptomatic
Karimi-Galougah et al. 2020 [8]	Iran	Letter to Editor	1	22	M	Left-sided SSNHL	L: Normal downsloping to severe SSNHL R: NR	Dyspnea, cough
Karimi-Galougah et al. 2020 [8]	Iran	Letter to Editor	1	40	F	Right-sided SSNHL, tinnitus	R: Moderate downsloping to severe SSNHL L: NR	Dyspnea, cough, malaise
Karimi-Galougah et al. 2020 [8]	Iran	Letter to Editor	1	23	F	Left-sided SSNHL, tinnitus, vertigo	R: NR L: Flat mild to moderate SSNHL	Dyspnea, cough

Regarding COVID-19 treatment, one (14%) patient was treated with hydroxychloroquine, one (14%) was treated with cholecalciferol, doxazosin, fluticasone nasal spray, folic acid, lansoprazole, loratadine, ramipril, rivaroxaban, tadalafil, and a salbutamol inhaler. Four (57%) patients were treated with intravenous and/or oral steroids intended to treat the SSNHL (Table 2). 

**Table 2:** Treatment and Treatment Outcomes of Patients With SSNHL and Confirmed COVID-19 Diagnosis N= Number of patients; F= Female, M=Male; NR = Not reported; SSNHL = Sudden sensorineural hearing loss; L: Left; R: Right

Author, Year	Comorbidities	COVID-19 Treatment	SSNHL Treatment	Treatment Outcome
Chern et al. 2020 [7]	NR	None	Prednisone, Intratympanic dexamethasone injection, oral steroid	Near-resolution of vestibular symptoms; Significant improvement in balance
Koumpa et al. 2020 [20]	Asthma	Colecalciferol, doxazosin, fluticasone nasal spray, folic acid, lansoprazole, loratadine, ramipril, rivaroxaban, tadalafil, salbutamol inhaler	Prednisolone, Intratympanic methylprednisolone sodium succinate injection	R: No audiogram reported L: Pure tone audiogram - 2, 3, 4 and 6 kHz with hearing thresholds of 55, 60, 60 and 80 dB respectively
Lang et al. 2020 [9]	NR	None	Oral prednisolone	No improvement on audiological assessment a week after prednisolone treatment
Kilic et al. 2020 [18]	NR	Hydroxychloroquine	Prednisolone, vitamin B, folic acid complex, proton pump inhibitor	Complete resolution of hearing loss
Karimi-Galougah et al. 2020 [8]	NR	NR	NR	NR
Karimi-Galougah et al. 2020 [8]	NR	NR	NR	NR
Karimi-Galougah et al. 2020 [8]	NR	NR	NR	NR

Discussion

COVID-19 has a wide variety of presentations, which can lead to a delay in diagnosis. In our study, patients presented with SSNHL as well as other symptoms including tinnitus, vertigo, and aural fullness. In addition, one of the patients also had a diagnosis of intralabyrinthine hemorrhage made via MRI [7]. Interestingly, SSNHL in the patients in our study presented at various points in the disease course. In Kilic et al., the patient’s only presenting COVID-19 symptom was SSNHL [18]. In Chern et al. [7], the otologic symptoms presented first, with a delayed onset of anosmia and ageusia, which are known to be common COVID-19 symptoms [20]. In Lang et al., SSNHL developed after the resolution of other COVID-19 symptoms [9]. In Koumpa et al., the patient had a complicated course of COVID-19 that involved intubation with resultant ventilator-associated pneumonia, pulmonary hypertension, bilateral pulmonary emboli, and anemia. He experienced SSNHL and tinnitus one week after extubation [19]. The delay in diagnosis due to atypical presentations, such as those reported, is problematic as permanent hearing loss has been associated with poor outcomes in regards to health and quality of life. The literature reports that permanent hearing loss is significantly associated with dementia, depression, and cognitive impairment [21]. In addition, tinnitus, which was reported in four of our seven cases, has been reported to be associated with impaired sleep and attention dysfunction [22].

To date, there has been no definitive evidence of the cause of SSNHL in patients with COVID-19. Like other viral infections, COVID-19 may cause SSNHL through direct viral invasion of cells in the peripheral and central nervous system via the angiotensin-converting enzyme 2 (ACE2) receptor [23]. This viral mechanism would allow COVID-19 to invade the auditory cortex in the temporal lobe. Damage to the auditory cortex occurs through the viral promotion of excess cytokine release, direct invasion of the cochlear nerve causing neuritis, and invasion of the soft tissues of the cochlea causing cochleitis [24]. Current studies have pointed to a possible COVID-19 presence in the inner ear implicating a viral etiology in SSNHL. Mustafa et al. found that 20 positive COVID-19 patients exhibited damage to the hair cells of the cochlea as determined by transitory evoked otoacoustic emissions (TEOAE) amplitudes, despite a lack of auditory complaints [25]. This finding supports a possible viral mechanism to the SSNHL seen in COVID-19 patients, as inner ear damage was seen in the absence of symptoms. In our review, Karimi-Galougah et al. specifically illustrated the potential viral causation of SSNHL in COVID-19, as they excluded patients treated with any pharmacologic agents [8]. In our entire study, there were five out of seven patients diagnosed with SSNHL in the setting of a COVID-19 diagnosis without confounding by COVID-19 pharmacologic treatment. 

Although there is data to support a viral mechanism behind SSNHL in COVID-19 patients, it is important to recognize that pharmacologic ototoxicity may also play a role. This mechanism is not fully understood, but the ototoxicity is suggested to be related to the destruction of the cochlear sensory hair cells, a decline in neuronal population, and changes in supporting structures [26]. This potential mechanism for SSNHL has been cited in the literature with 11 reports of chloroquine ototoxicity and six reports of hydroxychloroquine ototoxicity [14]. Studies have implied that the treatment regimen of COVID-19, specifically hydroxychloroquine, causes pharmacologic ototoxicity in SSNHL. In the cases included in our systematic review, two of the patients were treated with drugs with potential otologic side effects. Despite the fact that all of the patients included in our study experienced SSNHL prior to COVID-19 treatment, caution should still be taken when prescribing pharmacologic ototoxic agents.

Currently, the incidence of SSNHL in the United States is 6000 new cases annually [27]. It is important to recognize, however, that there are multiple causes of SSNHL outside of a COVID-19 viral cause or pharmacological toxicity. Prior to the COVID-19 pandemic, from March 15, 2019, to May 31, 2019, Chari et al. found that there were 71 SSNHL cases diagnosed out of 4013 suspected cases (1.77%). During the COVID-19 pandemic, from March 15, 2020, to May 31, 2020, there were 13 diagnosed SSNHL cases out of 681 suspected cases (1.91%). None of these 13 patients diagnosed with SSNHL were found to be positive for COVID-19 [28]. It should be noted that SSNHL is probably multifactorial and whether COVID-19 has an effect on these factors is unknown.

Reviewing the published literature does not clarify whether SSNHL is associated with COVID-19 but it is unlikely that it is solely a pharmacological effect secondary to treatment. We hope that this study raises awareness of the possible presentation of SSNHL in patients with COVID-19. In addition, we hope to bring awareness to the need for cautious use of the COVID-19 treatment regimen with regards to the possible ototoxicity of these pharmacologic agents. Further research in the field is needed to identify the causative mechanism behind SSNHL in patients with a diagnosis of COVID-19.

Strengths and limitations

Although there are other systematic reviews in the literature with a focus on otologic dysfunction seen in COVID-19 patients, our study is the first to identify COVID-19 patients with SSNHL specifically. In addition, we expanded our results and discussion to include treatment regimens, specifically assessing the correlation between COVID-19 treatment and SSNHL. There are currently no reports in the literature assessing how or if COVID-19 treatment regimens may correlate with the SSNHL specifically seen in these cases, despite the fact that hydroxychloroquine is a known ototoxic agent. The main limitation of our study is the sample size of seven total cases and the lack of clarity in the description of the cases, particularly in the details of the audiograms. Research with larger populations needs to be done to determine if there is a correlation between SSNHL and COVID-19 infection. In addition, further research needs to be conducted to determine whether the cause of the SSNHL is the novel coronavirus itself, the COVID-19 treatment regimens, or another unknown cause.

## Conclusions

Currently, there is variation in presentation of COVID-19. There are a small number of patients with COVID-19 infection and concurrent SSNHL, but presentation and clinical course vary. The literature suggests multiple possible mechanisms for SSNHL in COVID-19 patients. While all of the patients in our study reported SSNHL, not all of the patients were treated with ototoxic pharmacologic therapies. It is still possible that SSNHL presenting in COVID-19 patients represents the baseline incidence of SSNHL and is not directly caused by COVID-19. The current literature is insufficient to characterize the pattern of hearing loss or advise about the treatment or outcomes. Future studies require a larger database or population study.
